# Association Between Human Embryo Culture Conditions, Cryopreservation, and the Potential Risk of Birth Defects in Children Conceived Through Assisted Reproduction Technology

**DOI:** 10.3390/medicina61071194

**Published:** 2025-06-30

**Authors:** Romualdo Sciorio, Luca Tramontano, Giuseppe Gullo, Steven Fleming

**Affiliations:** 1 Fertility Medicine and Gynaecological Endocrinology Unit, Department Woman Mother Child, Lausanne University Hospital, 1011 Lausanne, Switzerland; 2 Département de Gynécologie-Obstétrique, Réseau Hospitalier Neuchâtelois, 2000 Neuchâtel, Switzerland; luca.tramontano30@gmail.com; 3 Azienda Ospedaliera Ospedali Riuniti (AOOR) Villa Sofia Cervello, IVF Public Center, Department of Medicine, University of Palermo, 90133 Palermo, Italy; gullogiuseppe@libero.it; 4 Discipline of Anatomy & Histology, School of Medical Sciences, University of Sydney, Sydney, NSW 2006, Australia; blueyfleming@gmail.com

**Keywords:** culture media, pH and osmolality, temperature, oxygen tension, cryopreservation and vitrification, birth defects, offspring health, epigenetic alterations, assisted reproduction technology (ART)

## Abstract

Assisted reproduction technology (ART) has advanced significantly over the past four decades, leading to improved pregnancy outcomes and a reduction in complications, particularly those associated with multiple pregnancies. These improvements largely stem from advances in understanding embryonic physiology, which has enabled better culture conditions. As a result, embryologists can now efficiently culture embryos to the blastocyst stage and successfully cryopreserve them for future use. However, while incubators aim to replicate the maternal environment of the oviduct and uterus, embryos in vitro are cultured in static conditions, unlike the dynamic, constantly changing environment they experience in vivo. Key factors such as pH, temperature, osmolality, and gas concentrations are crucial for establishing optimal embryo development and implantation potential. Moreover, the vitrification procedure for gametes or embryos can introduce oxidative stress, as well as osmotic shock and cryoprotectant toxicity, which may affect embryo viability and increase the risk of birth defects. Since the first successful ART birth in 1978, over 10 million babies have been conceived through these techniques. Although most of these children are healthy, concerns exist about potential birth defects or changes linked to the handling of gametes and embryos. The preimplantation period is marked by significant epigenetic reprogramming, which can be influenced by ART procedures such as ovarian stimulation, in vitro fertilization, embryo culture, and cryopreservation. However, the long-term health implications for offspring remain uncertain. Epigenetic reprogramming during early embryogenesis is essential for proper embryo development and can be changed by ART-related conditions. These concerns have raised questions about the possible connection between ART and a higher risk of birth defects or other changes in children born through these methods. Therefore, we conducted a scoping review following PRISMA-ScR guidelines to map evidence on ART-related risks, including epigenetic and birth defect outcomes.

## 1. Introduction

Embryo culture aims to create an environment that supports embryo development while minimizing stress. Since the 1970s, in vitro fertilization (IVF) culture systems have evolved to improve embryo quality, enabling culture to the blastocyst stage and facilitating elective single embryo transfer (eSET), which has reduced multiple pregnancies while maintaining high success rates [1,2]. Despite these advances, in vitro culture conditions may not fully replicate the embryo’s natural environment. Suboptimal conditions could impair development, viability, and the potential for implantation. The in vitro culture process includes features that could elevate embryonic stress, such as media composition, plastic dishes, oxygen tension, temperature, pH, and osmolality [3,4]. These factors significantly impact embryo development, making it crucial to continuously improve culture techniques to minimize stress. IVF laboratories must ensure optimal conditions to support viable embryos with high implantation potential and the future health of children. Over the past 40 years or more, assisted reproduction technology (ART) has enabled millions of infertile couples to conceive, resulting in over 10 million children being born. In some European countries, approximately 5% of births are ART-related [5,6,7]. While infertility remains the main reason for IVF use, an increasing number of individuals are opting to freeze oocytes [8,9,10,11] or embryos for future use, with nearly 310,000 frozen embryo transfer (FET) cycles in Europe in 2018. Although ART is generally considered safe, concerns have been raised about its association with low birth weight, birth defects, and metabolic disorders, potentially linked to epigenetic dysfunction in gametes and embryos [12,13,14]. As FET cycles increase, understanding their impact on future offspring health is crucial, particularly regarding potential epigenetic modifications [15,16,17,18,19]. The percentage of children born following ART is growing and currently is about 3–5%. It is essential to assess the potential negative effects of the ART procedure on the conceived baby. Epidemiological studies have indicated a higher incidence of low birth weight in children conceived through ART with fresh embryo transfer [20]. A similar finding was reported by Sunkara and colleagues, who analyzed data from the UK registry (Human Fertilization and Embryology Authority, HFEA) covering 1991 to 2016 and including approximately 117,000 singleton live births after ART. Their study showed that infertility negatively affects preterm birth and low birth weight following fresh embryo transfer [21]. In contrast, research on frozen-thawed embryo transfers (FETs) in ART has yielded different results. A large study by Terho and colleagues found that FETs are linked to higher birth weight and a greater risk of large-for-gestational-age (LGA) infants [22]. Therefore, this scoping review aims to systematically map and synthesize evidence on ART procedural risks, prioritizing breadth over quantitative synthesis.

## 2. Historical Landscape of Human Embryo Development

In vivo, during the first three days of embryonic development, the embryo travels through the oviduct. By days 4–5, it reaches the uterine cavity, undergoing compaction and blastocyst formation. In the early days of ART, scientists were typically using basic culture media, with embryo transfer at the cleavage stage, when embryos have about 4–8 cells [1]. Before embryonic genome activation (EGA), the mammalian embryo is transcriptionally silent and relies on maternal mRNA for its metabolic needs. EGA in human embryos occurs at around the 4- to 8-cell stage, and this signals a metabolic switch for its energy requirements [23,24]. In 1988, Braude and colleagues established that EGA in human embryos correlates with transcriptional activation and protein synthesis. At this stage, embryos predominantly use pyruvate and lactate for energy. After EGA, they switch to glucose-based metabolism [25]. Following genome activation, the embryo undergoes compaction, during which blastomeres tightly adhere to one another to form a cluster known as the morula. This marks the onset of radial symmetry. The morula forms a blastocoel cavity due to fluid secretion, which raises the salt concentration within the embryo and draws in water via osmosis. The blastocyst expands, thinning the zona pellucida (ZP), eventually leading to the hatching process. At this point, the blastocyst consists of the inner cell mass (ICM) and surrounding trophectoderm (TE) cells. The ICM develops into the early epiblast, which forms all foetal tissues, while the TE forms the placenta [1,26]. Implantation in humans occurs around day 7 of development. Failure of implantation, often caused by poor embryo development or uterine receptivity, is a significant barrier to ART success. Embryo viability is influenced by the composition of culture media. The introduction of advanced media has revolutionized ART, enabling embryos to grow beyond the cleavage stage. In the late 1990s, with more consistent media, embryo culture was extended to the blastocyst stage, improving pregnancy rates and reducing multiple pregnancies when transferring a single blastocyst [26,27,28,29,30].

### Evolution of Embryo Culture Media

**Figure 1:**
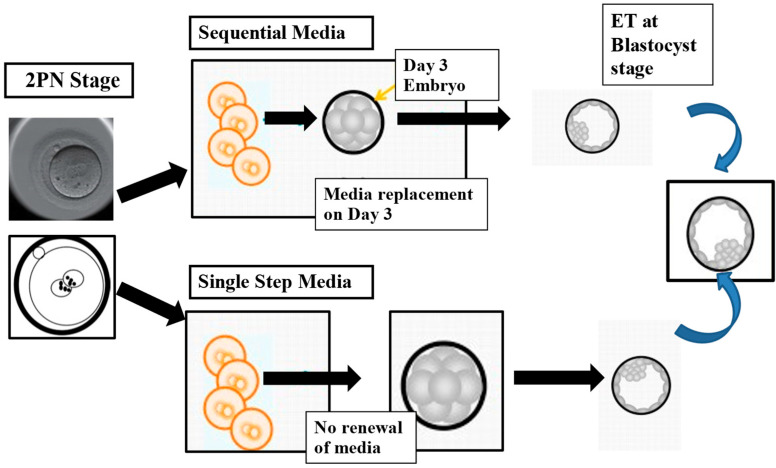
The illustration compares two methods commonly used in embryology labs for culturing human embryos: the sequential media approach, which mimics the natural changes in the reproductive environment by using different media at various developmental stages, and the single-step method, which maintains embryos in a consistent medium throughout development, from fertilization (2PN stage) to blastocyst formation. Modified with permission from Sciorio and Rinaudo [39].

In the 1940s, Rock and Menkin made early attempts at in vitro fertilization, collecting oocytes during laparotomy procedures [31]. Although their efforts to fertilize human oocytes were unsuccessful, they laid the groundwork for later advancements. In 1973, Landrum Shuttle claimed to have fertilized human eggs, but the embryos were discarded, drawing considerable media attention [32]. The first true IVF breakthrough came in 1978 with the birth of Louise Brown, thanks to the pioneer work performed by Edwards and Steptoe. Initially, IVF culture media were prepared “in-house,” based on simple media with several chemical compounds added, including patients’ serum, bovine serum albumin (BSA), penicillin, sodium pyruvate, phenol red, and bicarbonate. Embryos leading to the birth of Louise Brown were cultured in Earle’s basic salt solution enriched with pyruvate and serum from the patient [33,34,35]. In the 1980s, commercial media were introduced, typically based on modified versions of Earle’s balanced salt solution. Progress continued with Menezo and co-workers proposing the addition of amino acids (AAs) to the media, improving embryo growth [36]. Patrick Quinn designed the Human Tubal Fluid (HTF) medium to better support human embryos [37]. As knowledge of embryo metabolism advanced, particularly based on the transition from pyruvate and lactate usage at the cleavage stage to glucose metabolism post EGA, new culture media were developed to better mimic the physiological environment of the reproductive system. These so-called “sequential” media aimed to replicate the molecule and energy concentrations found in the female reproductive tract. Therefore, several companies started to introduce worldwide this new concept of sequential culture media, including SAGE in the U.S., MediCult/Origio and Scandinavian IVF Science/Vitrolife in Europe, and Cook in Australia. Those sequential media were based on the concept of a media change on day three to align with the embryo’s metabolic shift. In the early 1990s, Lawitts and Biggers [38] introduced a new concept for embryo culture, the “simplex optimization medium” approach, where embryos were cultured continuously in a single medium, from fertilization to the blastocyst stage, without needing to change the culture medium at any point (Figure 1) [38,39,40]. An advantage of using this method is that it reduces stress on the embryos. Moving embryos between different media can be stressful, and the transition itself can negatively affect development. This approach helps optimize embryo viability, making it a significant advancement in ART.

## 3. Media Composition and Embryo Development

Various factors can significantly influence embryo development; here, we examine how specific conditions may hinder the progression of human embryos. Currently, a variety of culture media are available on the market, each with different chemical compounds, AAs, protein sources, and macromolecules. However, the exact concentrations of the components in single-step or sequential media are kept secret by manufacturers, as media compositions cannot be patented and are considered trade secrets [41]. All media typically contain lactate, pyruvate, and glucose as carbohydrate sources in varying concentrations. Glucose is crucial for glycolysis and serves as a precursor for the synthesis of lipids, nucleic acids, and other biomolecules. A key unresolved question is whether one culture medium is superior to another in supporting embryo development, implantation, or live birth rates. This issue remains inconclusive due to limitations in many comparative studies, such as insufficient statistical power, suboptimal experimental design, or failure to control for confounding variables [42,43,44]. For example, some studies reported blastocyst formation rates without distinguishing between development on days 5, 6, or 7 [45]. A systematic review of randomized controlled trials published between January 1985 and July 2012, conducted by Mantikou and colleagues, examined clinical outcomes such as embryo quality, clinical pregnancy, miscarriage, and live birth rates [42]. The review found that due to poor study design, a conventional meta-analysis was not possible. Only four studies declared their live birth rates, and only one showed a significant difference between media [46]. Similarly, ongoing or clinical pregnancy rates were reported in nine trials, with four showing significant differences [47]. Overall, this analysis did not identify a clearly superior culture medium. Finally, while there is no definitive evidence that any culture medium is superior, further and well-designed studies are needed to answer this question.

### 3.1. Amino Acids and Protein Supplementation 

Culture media play critical roles beyond providing energy to developing embryos. One key aspect is the composition of AAs, which regulate various processes in mammalian embryo development. AAs function as metabolites, antioxidants, pH buffers, osmolality regulators, and heavy metal chelators [39,48]. Specific AAs, such as glutamine, and non-essential AAs (e.g., alanine, asparagine, aspartate, glutamate, glycine, proline, and serine) support early embryo development, while both essential and non-essential AAs contribute to the growth of the ICM [48]. Non-essential AAs are involved in TE stimulation and ZP hatching [49]. Mouse studies suggest that limiting AAs can impair embryo development, emphasizing the necessity of including AAs in culture media [50]. Menezo and collaborators investigated how methionine is involved in crucial pathways, including glutathione, hypotaurine, and taurine pathways, influencing chromosomal stability through processes such as imprinting and DNA methylation [51]. A study by Clare and colleagues performed in bovine embryos demonstrated that reduced methionine levels could lead to DNA methylation in over 1600 genes, including several imprinted genes linked to an abnormal foetal overgrowth phenotype [52]. Another concern with in vitro culture is that the addition of AAs can increase ammonium production. At 37 °C, AAs degrade over time, leading to the accumulation of ammonium, a compound toxic to embryos that can impair implantation and adversely affect fetal development. The impact of ammonium is intensified when culture is performed at 20% oxygen tension [53]. A solution to this issue is to use more stable dipeptide forms, such as alanyl-l-glutamine or glycyl-l-glutamine, which significantly reduce ammonium accumulation and create a safer environment for embryo development [48,50,54,55]. Another important compound of culture media is represented by the addition of human serum albumin (HSA) and complex protein supplements, enhancing embryo development and increasing live birth rates [56]. HSA, prevalent in the oviduct, offers multiple benefits, including preventing embryo adhesion to consumables, stabilizing membranes, and providing nitrogen sources, pH buffers, and chelation of trace elements and toxins [56,57,58,59]. However, protein supplements may contain unwanted additives, such as preservatives and growth factors, which can negatively affect embryo development, as seen with octanoic acid, which contains toxic pro-oxidant metals [60].

### 3.2. pH and Osmolality

The pH of culture media is critical for embryo development, as it directly affects embryo metabolism, viability, and growth. The pH of the culture medium is primarily influenced by its bicarbonate content and the CO_2_ tension of the incubator, along with external factors such as media composition, laboratory conditions, and altitude. Variations outside the physiological pH range can impair embryo development, as shown in mouse studies linking abnormal pH with foetal growth issues [61]. Embryos can adapt to changes in pH, but oocytes are more vulnerable due to their limited internal pH regulation [62]. Therefore, maintaining stable pH within the physiological range during oocyte retrieval and embryo culture is essential for optimal development [63]. For this reason, additives such as zwitterionic buffers, including MOPS and HEPES, are used to stabilize pH when gametes and embryos are outside the incubator. These buffers are considered safe and help maintain pH consistency [63,64,65]. Historically, phenol red was used to indicate pH changes visually, but it has been linked to reactive oxygen species (ROS) generation and potential estrogenic effects. Consequently, various modern media formulations have removed phenol red [66]. Additionally, another critical feature affecting human embryo development is the medium osmolality. Elevated osmolality can negatively affect cell volume, cell surface, and membrane stability, inducing stress and inhibiting embryo development, as shown in mouse studies. Osmolality is influenced by the media’s chemical composition, including proteins and AAs. Early embryo stages are particularly sensitive to osmolality changes, as cell volume homeostasis is vital for development. Mammalian embryos develop best at an osmolality of 255–295 mOsm/kg, while values above 300 mOsm/kg can cause osmotic stress and reduced implantation potential [67,68,69,70]. While commercial media manufacturers set media osmolality, laboratory factors such as evaporation, culture dish preparation time, media droplet volume, oil overlay, incubator humidity, airflow, and temperature can lead to variation. Maintaining consistent osmolality is crucial, and thus, strict adherence to laboratory protocols is essential for optimal embryo development [69]. Finally, to better maintain physiological levels of pH, osmolality, and temperature during embryo development, novel benchtop incubators, including those with time-lapse technology, are considered better than large incubators for stable culture conditions [69,70,71].

### 3.3. Temperature

Temperature is extremely important for a variety of aspects relevant to gamete and embryo physiology, including metabolism and the stability of the meiotic spindle (MS). The MS is a structure that helps segregate chromosomes correctly during meiosis and is considered a key indicator of oocyte health [72]. Its stability is linked to fertilization, zygote division, and chromatin segregation, with any instability leading to chromosomal errors, aneuploidies, implantation failure, and miscarriage [73]. The MS is sensitive to changes in temperature and pH, and outside the physiological range, it becomes unstable. Studies have shown that the MS begins to disassemble at temperatures below 33 °C, with prolonged exposure to non-physiologic conditions resulting in complete depolymerization of the spindle. Both animal and human studies have highlighted the negative effects of temperature, pH, and osmolality fluctuations on microtubule stability and spindle function [72,73,74,75,76]. Research has shown that keeping the temperature between 35 °C and 37 °C during oocyte recovery is beneficial for embryo development in bovine and mouse models [76]. Similarly, a stable temperature while manipulating human oocytes improves fertilization rates. Generally, 37 °C is generally considered optimal for embryo culture, as it mimics the natural in vivo body temperature. However, human body temperature fluctuates depending on factors such as metabolism, diet, sex, time of day, and the body area measured. For instance, during the luteal phase, the female body temperature increases, with the oviduct and follicle being cooler than the body by about 1.5 °C and 2.3 °C, respectively [77]. While there is limited research on how temperature variations affect embryo development in vitro, some studies have explored this. For example, De Munck and colleagues compared a stable 37 °C with a fluctuating temperature range (36.6 °C to 37.5 °C) and found no significant differences in fertilization rates, embryo quality, or live birth rates [78]. A study by Fawzy and co-workers, involving 412 women, compared culture at 37 °C and 36.5 °C and found no significant differences in pregnancy or implantation rates [79]. However, the cooler temperature of 36.5 °C was linked to a higher cleavage rate but reduced fertilization, fewer high-quality embryos on day 3, and lower blastocyst formation on day 5. In another study by Hong and co-authors, human embryos cultured at 37 °C showed higher blastocyst formation rates compared to those at 36 °C, though other metrics such as fertilization and implantation were similar [80]. These findings suggest that while embryos have some ability to adapt to temperature changes, in vitro culture should ideally occur at 37 °C for optimal pregnancy outcomes in ART.

## 4. Oxygen Tension and Oxidative Stress

Oxygen concentration plays a critical role in human embryo development and metabolism, as it impacts both therapeutic benefits and potential harm. During mitochondrial oxidative phosphorylation, oxygen is consumed, and ROS may be produced due to the leakage of high-energy electrons in the electron transport chain. These ROS can impair cellular metabolism, compromise DNA integrity, and reduce embryo viability. Culturing embryos in atmospheric oxygen (around 20%) increases ROS generation. However, in mammals, the natural oxygen levels within the female reproductive tract range approximately 2% to 8%, indicating that embryos are not naturally exposed to such high oxygen levels in vivo [77,81,82]. Therefore, incubating embryos at a reduced oxygen level, typically around 5%, is widely recommended. Studies in animal models such as rats, mice, cats, sheep, and pigs have demonstrated improved outcomes with lower oxygen concentrations during embryo culture [77,81,82,83,84,85,86]. Similarly, human research suggests that reduced oxygen environments may enhance embryo development, as well as improve pregnancy and live birth rates. Further reports have shown that atmospheric oxygen can negatively affect embryos, altering gene expression, protein function, and metabolism [77,82,83,84,85,86]. A randomized controlled trial by Meintjes and collaborators demonstrated that culturing human embryos at reduced oxygen levels significantly increased pregnancy, implantation, and live birth rates [85]. These findings have been confirmed by several studies [77,81,82,83,84,85,86]. Although the exact mechanisms remain unclear, researchers hypothesize that a benefit of low oxygen can be associated with a reduction in ROS and improved air quality by reducing volatile organic compounds, resulting in better embryo gene expression and epigenetic profiles [15,16,86,87]. ROS can cause damage to cell organelles, including DNA fragmentation, protein dysfunction, and lipid damage [88]. Mitochondria are also affected by oxidative stress [61,89]. A study on mouse embryos found that IVF-generated embryos cultured at 20% oxygen had fewer mitochondria and more abnormal mitochondria compared to embryos generated through spontaneous mating [86]. Oxidative stress can also alter embryonic epigenomes [86,90]. Li and colleagues examined the effects of high oxygen levels (20%) in bovine embryos and found a significantly increased DNA methylation in cleavage and blastocyst stage embryos [91]. Additionally, high oxygen tension affected histone marks in bovine blastocysts and altered the embryo proteome in mice [92]. Culturing embryos at high oxygen led to the downregulation of proteins and abnormal gene expression [86,93]. In conclusion, culturing embryos at low oxygen concentrations promotes faster development and less disruption in gene expression. This method is now the preferred practice in clinical settings, with most IVF laboratories using 5% oxygen [90,91,92,93,94]. However, according to a study by Christianson and colleagues, albeit as early as 2014, they reported that only 25% of IVF laboratories worldwide exclusively used 5% oxygen [95].

## 5. Cryopreservation and Cryoprotectants

**Figure 2:**
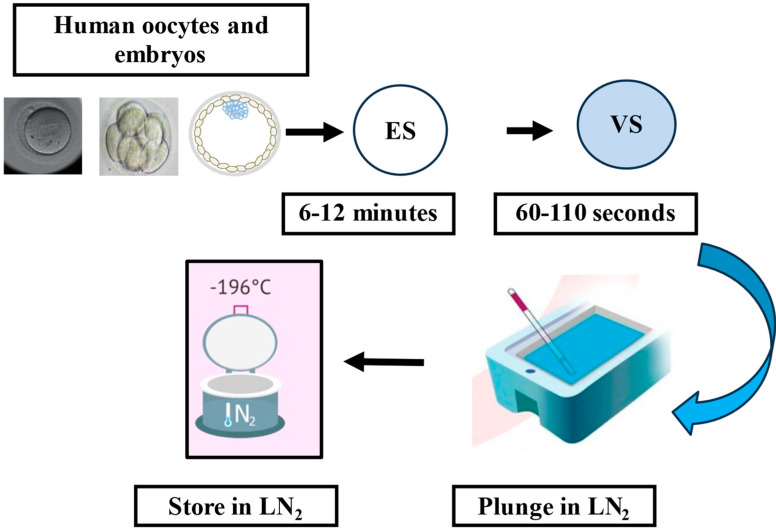
The diagram illustrates the cryopreservation procedure primarily used for preserving human oocytes and embryos, known as the vitrification method. Initially, the embryo or oocyte is placed in an equilibration solution for up to 12 min to initiate the dehydration process. This is followed by the vitrification step, during which the embryo or oocyte is exposed to a highly concentrated cryoprotectant solution for up to 60 s before being plunged into liquid nitrogen. ES: equilibration solution, VS: vitrification solution, LN_2_: liquid nitrogen.

Cryopreservation involves freezing cells or tissues and storing them in liquid nitrogen (LN_2_) at −196 °C, halting all biological activity while maintaining cell viability for future use (Figure 2). This method has been widely applied in ART to preserve human gametes and embryos. The first successful live birth from a thawed cryopreserved embryo using the “slow freezing” method was reported in Australia in 1983 by Trounson and Mohr [96]. In the 1990s, vitrification emerged as a significant improvement, offering better survival and pregnancy outcomes compared to slow freezing [9,97]. Vitrification has since become the preferred method for cryopreserving human oocytes and embryos, with evidence showing superior results over slow freezing protocols [9,98,99,100]. The success of vitrification depends on several factors, including the temperatures used during vitrification and warming, the type of carrier (open or closed vitrification) and, most importantly, the concentration and type of cryoprotective agents (CPAs) used. Studies have reported that the warming rate is as crucial as the cooling rate. Seki and Mazur found that improper warming, due to re-crystallization, can cause damage, and they concluded that a warming rate of at least 3000 °C/min is essential to maintain oocyte cryo-survival rates above 80% [101]. CPAs protect cells during the freezing process by preventing cryo-damage. There are two main types of CPAs: penetrating and non-penetrating. Penetrating CPAs, such as dimethyl sulfoxide (DMSO), ethylene glycol, and glycerol, have small molecular weights and can pass through cell membranes to protect the cells from freezing injury. Non-penetrating CPAs, such as trehalose, sucrose, and glucose, cannot cross the cell membrane but create an osmotic gradient that helps reduce ice formation by drawing water out of the cell [102]. However, CPAs are not without risks, as they can cause cellular toxicity, which depends on factors including exposure time, temperature, and concentration. Efforts to minimize CPA toxicity have focused on reducing exposure times and temperatures [103,104]. A recent approach by Liebermann and colleagues proposed ultra-fast warming, where embryos are immersed in a thawing solution for just one minute at 37 °C before being transferred to culture media. This method reduces time outside the incubator, minimizing oxidative stress and improving cryo-survival, implantation, and pregnancy rates [105].

### Application of Cryopreservation Procedures in ART

**Figure 3:**
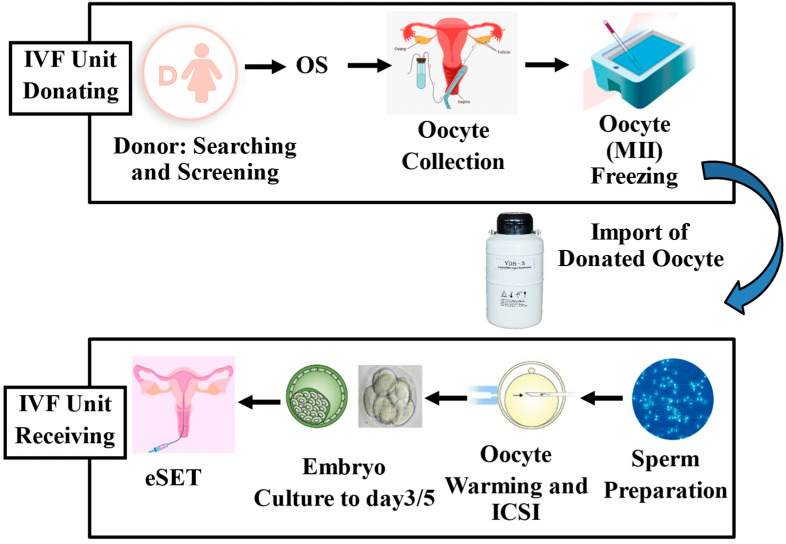
The illustration depicts the imported oocyte donation program, which utilizes cryopreserved oocytes obtained from a donor bank. In this process, oocytes are collected and vitrified in the donor bank laboratory. Once cryopreserved, they are carefully transported to the recipient clinic, where they are warmed and subsequently used in fertility treatment for the intended recipient. eSET, elective single embryo transfer; ICSI, intracytoplasmic sperm injection; MII, metaphase II oocyte; OS, ovarian stimulation. Reprinted with permission from Sciorio et al., 2023 [104].

Over the past few decades, advancements in ART have led to significant improvements in cryopreservation techniques for both human embryos and oocytes. In the USA alone, it is estimated that 600,000 embryos were stored between 2004 and 2013, and in Europe, 335,744 FETs were performed in 2019 [6]. Cryopreservation serves a variety of purposes, including storage of surplus embryos after fresh transfers, support of the eSET policy, preservation of fertility for cancer patients, and enablement of pre-implantation genetic testing [106,107,108,109]. Other purposes include management of abnormal stimulation cycles (e.g., elevated progesterone levels) and prevention of ovarian hyperstimulation syndrome, a serious complication [105,106,107,108]. Current evidence indicates that ART treatment, including cryopreservation procedures, is generally safe. However, some studies have reported associations between ART and a higher incidence of low birth weight, birth defects, altered growth, and metabolic disorders [12,110,111,112,113,114,115,116]. For example, FETs have been linked to increased birth weights compared to fresh transfers or natural conception in the absence of cryopreservation [112,113]. A meta-analysis of 26 studies found that singletons born after freezing and thawing had higher birth weight and were more likely to be LGA, with an increased risk of hypertensive disorders [114]. While it remains unclear whether vitrification, CPAs, placental development, or parental infertility contribute to this effect, studies have found no difference in birth weight when embryos are transferred during a natural cycle, suggesting that hormonal medications used for endometrial preparation might influence birth outcomes [115]. Nevertheless, advancements in cryopreservation techniques have significantly improved oocyte freezing, making it a valuable option for fertility preservation, especially for women postponing pregnancy or those whose fertility may be compromised by oncology treatment. The trend of delaying the first pregnancy has led to a growing demand for oocyte freezing. In the UK, elective egg freezing is the fastest-growing fertility treatment, with a 10% annual increase [117]. Over the past decade, egg-freezing cycles in Spain have grown from 4% to 22% of all vitrification procedures. In the USA, fertility preservation cycles increased from 9607 in 2017 to 13,275 in 2018, reflecting a broader global trend [115,116,117,118]. Oocyte cryopreservation is particularly beneficial for young cancer patients, whose fertility may be compromised by medical treatments [106,119,120]. In 2020, there were an estimated 19.3 million new cancer cases globally, with breast cancer being the most common diagnosis [121]. Oocyte cryopreservation is also used in egg donation programs (Figure 3), which have expanded significantly in the last few years. The number of oocyte donation cycles in the USA, for example, increased from 10,801 in 2000 to 49,193 in 2017 [121]. Oocyte banks play a crucial role in this process, collecting and freezing eggs for later use in IVF procedures, including genetic testing or fertilization with fresh or frozen spermatozoa. Studies have shown that oocyte vitrification provides high survival rates after warming and yields pregnancy rates comparable to those using fresh donor oocytes [122,123,124,125].

## 6. Epigenetics and the Embryonic Epigenome

**Figure 4:**
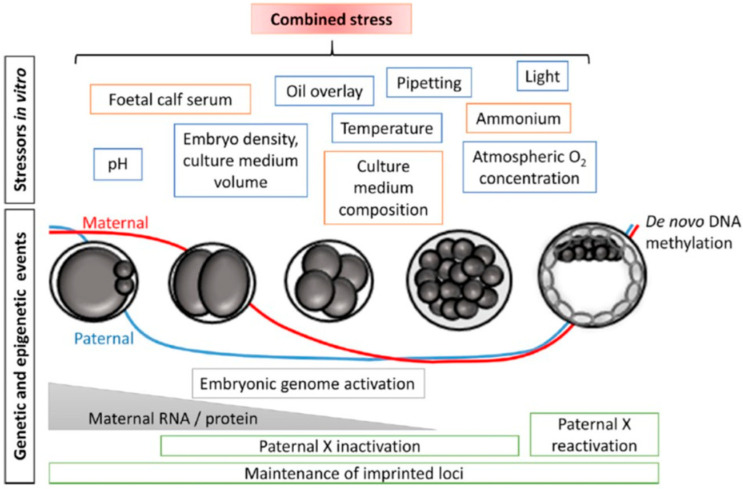
This summary outlines the critical genetic and epigenetic events that occur during preimplantation embryo development, with particular emphasis on the timing of the vitrification procedure. Multiple stressors may influence embryonic development, especially within the first five days, a period marked by intense cellular activity and rapid evolution. The illustration highlights specific epigenetic regulatory processes that are crucial for proper embryo development and successful implantation. These stressors can act synergistically, potentially amplifying adverse effects that may have long-term consequences for the health of the resulting child. Reprinted with permission from Sciorio and colleagues, 2023 [16].

In 1942, Conrad Waddington emphasized the importance of environmental interactions with genes during the early stages of embryo development. Despite the limited understanding of embryogenesis at the time, Waddington underlined the need to explore the factors that regulate developmental processes and mediate the relationship between genotype and phenotype. He introduced the term “epigenetics”, describing it as “the branch of biology that studies the causal interactions between genes and their products that bring the phenotype into being” [126]. This concept signaled a shift in understanding gene expression, not as solely dictated by the genetic code, but also influenced by external factors that impact development. Epigenetic regulation is essential for normal mammalian development, controlling gene activity without altering the DNA sequence (Figure 4) [127]. This regulation is responsible for controlling a variety of processes, from cell differentiation to the maintenance of tissue identity. In mammals, epigenetic changes occur in waves, resetting the epigenome in both germ cells and preimplantation embryos. The first wave of reprogramming occurs early in embryogenesis, when epigenetic marks are reset to prepare the embryo for further development. Notably, DNA methylation marks at imprinted genes, which are genes that are expressed in a parent-of-origin-specific manner, are retained during this phase. The second wave takes place during the development of primordial germ cells (PGCs) in the foetal gonadal ridge. Here, global DNA methylation marks are erased, including those at imprinted genes, resetting the epigenome in preparation for the next generation. After this erasure, parental imprints are re-established during germ cell differentiation, with distinct methylation patterns in male and female germ cells, ensuring proper gene expression in the offspring. During these stages of epigenetic reprogramming, the epigenome is particularly vulnerable to both environmental and internal factors that can alter the reprogramming process. Such disruptions may have long-term effects, including an increased risk of disease in future generations [128,129,130]. One of the most extensively studied epigenetic modifications is DNA methylation, which involves the addition of a methyl group to the 5’ carbon of the cytosine pyrimidine ring in CpG dinucleotides [131]. DNA methylation patterns are maintained through cell divisions by DNA methyltransferase 1 (DNMT1) [132], ensuring the stability of epigenetic modifications. Disruptions to these modifications during critical developmental windows can result in improper gene expression, leading to developmental disorders and an increased risk for a range of diseases later in life, including cancer, neurological disorders, and metabolic conditions [132,133,134]. This intricate balance between genetic information and epigenetic regulation highlights the importance of understanding how external influences, such as diet, toxins, or stress, can affect gene expression. Waddington’s work highlighted the dynamic relationship between genes and the environment, emphasizing that the correct development of an organism is not solely determined by its DNA sequence but also by epigenetic factors, which can be inherited and influenced by environmental conditions.

### 6.1. Potential Impairment by Vitrification and Epigenetic Alterations

In recent years, several research groups have explored the relationship between the cryoprotectants used during the vitrification procedure and epigenetic disruption in early embryo development during ART [135]. One of the most studied cryoprotectants is DMSO, commonly used to cryopreserve human embryos and gametes. DMSO can impair cellular function, metabolism, enzyme activities, cell growth, and apoptosis, and it may induce alterations in microRNAs (miRNAs) and epigenetic dysfunction [136,137]. Research has shown that DMSO exhibits toxic effects that vary depending on temperature, exposure duration, and concentration [138]. Investigations into the relationship between DMSO and epigenetic modification have indicated that DMSO may disrupt the function of the enzyme DNMT3a, although the precise underlying mechanism remains unclear [135,136,137]. Animal studies have demonstrated that vitrification and warming of mouse oocytes can significantly reduce the expression of the imprinted gene *Kcnq1ot1* [139]. In another study, Chen and colleagues observed that vitrifying mature bovine oocytes led to abnormal increases in the expression of the imprinted genes *Peg10*, *Kcnq1ot1*, and *Xist* in blastocysts generated following ICSI [140]. Follow-up research by the same group revealed that vitrification of mouse MII oocytes altered the expression of the maternally imprinted genes *Peg3*, *Peg10*, and *Igf2r* in oocytes and the paternally imprinted gene *Gtl2* in cleavage-stage embryos [141]. Further studies have reported reduced methylation levels of imprinted genes such as *H19*, *Peg3*, and *Snrpn* in mouse blastocysts derived from vitrified mouse oocytes [142]. Similar findings suggest an overall decrease in global DNA methylation levels in oocytes and early embryos following vitrification [143,144,145,146].

### 6.2. Human Studies

Human studies on the effects of vitrification on epigenetic regulation are limited due to challenges in obtaining research material and ethical concerns (Table 1). However, some studies have examined the effects of DMSO on DNA methylation. For instance, research on human cardiac microtissues revealed dysregulation of DNA methylation pathways, with increased expression of methyltransferases DNMT1 and DNMT3A, critical for maintaining DNA methylation, while *TET1*, which has an important role in active demethylation, was downregulated [137]. Despite these findings, studies on human oocytes and embryos following vitrification have reported minimal or no significant changes in DNA methylation or imprinted gene expression. In one investigation, the imprinted genes *H19* and *Kcnq1ot1* showed no differences in DNA methylation in vitrified oocytes [147]. In this study, immature oocytes donated after egg retrieval were vitrified and later in vitro matured to the MII stage [147]. Liu and colleagues investigated the effects of vitrification on nuclear configuration and global DNA methylation in germinal vesicle (GV)-stage oocytes, which were vitrified, warmed, and then matured to the MII stage. While they observed no significant differences in mitochondrial distribution or global DNA methylation patterns, a significantly higher rate of abnormal spindle configuration was noted following vitrification [148]. Similarly, De Munck found no notable changes in overall DNA methylation level in 8-cell embryos derived from vitrified oocytes [149]. In another study, Huo and colleagues analysed 16 donated human MII oocytes and identified 1,987 genes that were differentially expressed after oocyte vitrification and warming, with 82% of genes downregulated and 18% upregulated [150]. These genes were involved in various critical biological processes, including meiosis. For instance, key meiotic genes such as *Ncapd2* and *Tubgcp5* were significantly downregulated after vitrification [150]. An important consideration is whether the length of storage in LN_2_ could lead to epigenetic changes. Studies have generally found no alteration in gene expression associated with the duration of storage, suggesting that any damage observed after vitrification is more likely due to the cryopreservation process itself rather than storage duration [150,151,152]. This finding was confirmed by Stigliani and colleagues, who found no difference in gene expression between oocytes stored for three or six years in LN_2_ [152]. Similarly, research by Yan and collaborators on the impact of storage length on embryo survival and implantation showed that blastocysts stored for over six years had significantly lower survival, pregnancy, and live birth rates compared to those stored for less than three years, although no difference was observed in miscarriage or ectopic pregnancy rates [153]. To summarize, while animal models suggest that vitrification can affect imprinted gene expression and change the DNA methylation level [143,144,145,146], epigenetic changes in humans appear to be limited. The clinical significance of these changes remains unclear, and further research is needed to fully understand the potential consequence of vitrification on human oocytes and embryos [16,18,154,155,156].

## 7. Potential Risk of ART Procedures and Epigenetic Dysfunction

During early development, embryonic cells undergo a process in which they are directed toward their future cell types through epigenetic reprogramming and the restoration of cell-type-specific epigenetic marks. This critical period overlaps with the time when gametes and embryos are manipulated and cultured in the embryology laboratory during ART. As a result, such artificial interventions during this sensitive time can potentially cause epigenetic disruptions in the offspring that develop from these embryos. Several studies have highlighted that imprinted loci are particularly susceptible to environmental influences during embryo culture. For example, abnormal methylation patterns of *KvDMR1* have been observed in humans with Beckwith-Wiedemann Syndrome (BWS) following ART procedures, and hypomethylation of this locus has been found in bovine conceptuses derived from ART, which showed signs of Large Offspring Syndrome (LOS) [157,158,159,160,161,162]. Additionally, research has demonstrated that ART-related procedures, such as controlled ovarian stimulation, ICSI, and embryo manipulation, might lead to epigenetic abnormalities [157,159,163]. A systematic review by Lazaraviciute and co-authors [164] evaluated the incidence of imprinting disorders and DNA methylation changes at key imprinted genes in children conceived through ART compared to those conceived naturally. The review included 18 studies and reported a combined odds ratio of 3.67 (95% CI), indicating a higher incidence of imprinting disorders among ART-conceived children. The authors concluded that babies born via IVF and ICSI have an increased risk of imprinting disorders. However, the evidence linking ART to epigenetic alterations at specific imprinted genes was limited [164]. Another review, which summarized findings from eight studies focusing on BWS and ART, found a significant positive correlation between IVF/ICSI procedures and BWS, with an approximately 5.2-fold higher relative risk (95% CI 1.6–7.4) in children born through ART [165]. However, the authors did not observe an association for either Angelman Syndrome (AS) or Prader-Willi Syndrome (PWS) with IVF/ICSI, although a link was found between fertility problems and these conditions. Furthermore, the data on Silver-Russell Syndrome (SRS) was limited due to a small sample size (n = 13), and, therefore, no clear conclusions could be made regarding the incidence of SRS in ART-conceived children. Another epidemiological study conducted in Denmark and Finland assessed the risk of imprinting disorders in children conceived via ART [166]. The authors compared the incidence of PWS, SRS, BWS, and AS among ART-conceived children (*n* = 45,393 born 1994–2014 in Denmark and *n* = 29,244 born 1990–2014 in Finland). Their study reported a significantly increased odds ratio for BWS (OR 3.07, 95% CI: 1.49–6.31) in ART-conceived children, while no significant associations were found for PWS, SRS, and AS [166]. Similarly, a nationwide study in Japan found a 4.46-fold increase in BWS and an 8.91-fold increase in SRS in children born following ART, with many cases exhibiting abnormal DNA methylation at imprinted genes [167]. These findings underscore the growing recognition of how altered epigenetic marks and epimutations may influence human health, highlighting an important and evolving area of medical research. Further investigations, including large-scale national studies, need to be conducted to determine whether ART-induced epigenetic changes or the aforementioned syndromes are associated with specific patient characteristics or with the infertility conditions of both parents.

### ART Procedures, In Vitro Culture, and Birth Weight

Birth weight is a crucial metric related to fetal growth and is suggested by several authors as a potential prognostic factor of long-term risk of metabolic disease. Low birth weight is known to be associated with higher rates of coronary heart disease, as well as related disorders such as stroke, hypertension, and non-insulin-dependent diabetes [168,169]. Dumoulin and co-workers conducted a study comparing pregnancy rates and perinatal outcomes following 826 first IVF cycles, where embryos were randomly cultured in two different types of sequential media [170]. Among the 110 live-born singletons analyzed, a statistically significant difference in birth weight was observed between the two groups (3453 +/− 53 g versus 3208 +/− 61 g, *p* = 0.003), after adjusting for gestational age and gender. The authors concluded that the culture conditions used during in vitro development could influence birth weight in ART-conceived singletons [170]. This conclusion was later supported by a follow-up study from the same group involving a larger cohort of 294 live-born singletons [47]. Similar findings have been reported by other research groups [171,172,173,174]. Further evidence suggests that the type of IVF culture medium may also influence postnatal growth during the first two years of life, reinforcing the idea that early embryonic development is highly sensitive to its environment, with potential long-term consequences [175]. A comprehensive review by Lu and collaborators emphasizes that the majority of children conceived through ART are healthy. Nevertheless, growing evidence suggests that these children may face increased risks of low birth weight, lower gestational age, premature delivery, prenatal morbidity, as well as epigenetic disorders. The underlying mechanisms behind these outcomes remain incompletely understood [176]. Therefore, ongoing monitoring of children conceived via ART as they progress into adolescence and adulthood is crucial [176]. However, not all studies support these findings. For example, a retrospective analysis by Lin and co-authors found no significant difference in birth weight or length among newborns cultured in three different commercially available media [177]. Other independent studies also reported no meaningful differences in birth weight based on culture medium [178,179]. These conflicting findings have kept the debate ongoing, highlighting the need for more robust, long-term studies tracking the growth and health of ART-conceived children.

In addition to the type of culture medium, several other factors during in vitro culture may affect birth weight, including the age of the medium, its storage duration in the refrigerator or incubator [180], and variations in protein sources and concentration [181]. One of the most debated aspects is the timing of embryo transfer: whether it occurs at the cleavage stage (day 2–3) or the blastocyst stage (day 5–6). Zhu and colleagues addressed this in a retrospective study involving 2929 singletons, finding that those born after blastocyst transfer had significantly higher birth weights compared to those from day 3 embryo transfers (3465.31 ± 51.36 g versus 3319.82 ± 10.04 g; *p* = 0.009) [182]. A systematic review of 11 human studies exploring the link between culture media and birth weight found mixed results: six studies reported a significant impact on birth weight, while five found no effect [183]. As discussed earlier, epidemiological data indicate that fresh embryo transfers in ART are associated with an increased risk of low and very low birth weight [20,21]. In contrast, a different pattern emerges following FETs in ART. A large population-based study analyzed data from Denmark, Norway, and Sweden between the years 2000 and 2015, comparing birth weights of live-born singletons conceived after FETs (n = 17,500), fresh embryo transfers (n = 69,510), and natural conception (n = 3,311,588). The results showed that birth weight was significantly higher after FETs compared to fresh embryo transfer for both boys and girls [22]. Consistent findings were reported in the USA by Litzky and co-workers, who analyzed registry data from 2007 to 2014. In this study, FETs (n = 55,898) were associated with an average increase of 142 g in birth weight compared to fresh embryo transfers (n = 180,184; *p* < 0.001) [184].

## 8. Concluding Remarks

Currently, ART procedures have enabled millions of infertile couples to have children and are generally considered safe. However, concerns persist regarding the safety of these methods on the health and well-being of the offspring, both at birth and in later adult life. This review aimed to explore the potential risk of ART procedures, including in vitro culture and cryopreservation, regarding birth defects or epigenetic alterations following ART. Several animal studies and retrospective follow-ups of ART-born children suggest an increased risk of epigenetic errors, especially at imprinted loci. However, conclusive evidence linking ART to epigenetic modifications and long-term disease risk remains lacking. Notably, ovarian stimulation, manipulation of oocytes and embryos, and cryopreservation procedures should be restricted to a minimum to reduce potential negative effects. Unfortunately, many decisions in human ART are made without conclusive evidence, as long-term follow-up studies are still very limited. Therefore, large-scale epidemiological studies assessing the impact of ART on offspring health at birth and in adulthood are urgently required. Finally, future research using advanced technologies such as single-cell sequencing and epigenomics is essential to better understand the potential epigenetic aberrations occurring during oocyte and embryo manipulation or cryopreservation. This will help improve the safety and efficacy of ART procedures.

## Figures and Tables

**Table 1:** Summary of both human and animal studies showing the effects of vitrification on DNA methylation and histones modifications. GV; oocyte at germinal vesicle stage, MII; oocyte at metaphase II stage, IVM; in vitro maturation, 5hmC; 5-hydroxymethylCytosine, 5mC; 5-methylCytosine. DMR; differentially methylated regions.

Study [Ref]	Materials: Human or Animal	Oocytes or Embryo Analyzed (n)	Technology of Assessment	Studied Sequences or Genes	Main Findings
Al-Khtib et al., 2011 [147]	(Human) GV oocytes donated for research and IVM to MII	77 MII after IVM from 184 vitrified GV stage, and 85 MII from 120 fresh GV	Pyrosequencing	Methylation profile of *H19* and *KCNQ1OT1*, *H19-DMR*, and *KvDMR1*	Oocyte vitrification at the GV stage does not affect the methylation profiles of *H19-DMR* and *KvDMR1*
Zhao et al., 2020 [143]	(Bovine) Oocytes	Vitrified MII oocytes from matured in vitro	Single-cell whole-genome methylation sequencing	Global analysis	*Peg3* methylation level was significantly decreased in derived blastocysts
Cheng et al., 2014 [145]	(Mouse) Blastocysts	Blastocysts from Vitrified MII oocytes	Bisulfite sequencing	*H19*, *Peg3*, *Snrpn*	No significant differences in oocytes. Decrease in blastocysts after oocyte vitrification.
De Munck et al., 2015 [149]	(Human) Mature (MII) donated oocytes	31 embryos (Day-3) from 17 fresh oocytes and 14 after vitrification	Immunofluorescence (5mC, 5hmC)	Global Analysis	No differences in fluorescence intensities between embryos from fresh and vitrified oocytes
Liu et al., 2017 [148]	(Human) Vitrified mature oocytes (MII), and MII from GV matured in vitro	56 in vivo MII, 106 MII from GV matured in vitro, 122 MII from vitrified GV	Immunofluorescence (5mC)	Global analysis	No significant differences in fluorescence intensities between the groups
Barberet et al., 2020 [156]	(Human) Placenta	Review manuscript	Pyrosequencing and q-PCR	*H19*, *IGF2*, *KCNQ1OT1* *SNURF*	The placental DNA methylation levels of *H19/IGF2* were lower in the fresh embryo transfer group than in the control (*H19/IGF2*-seq1) and frozen embryo transfer (*H19/IGF2*-seq2) groups

## Data Availability

No new data were created or analyzed in this study.
